# Saudi Cerebral Palsy Register (SCPR): Protocol on the Methods and Technical Details

**DOI:** 10.1007/s44197-024-00198-5

**Published:** 2024-02-15

**Authors:** Sami Mukhdari Mushta, Riyad Alghamdi, Hani Almalki, Saadia Waqas, Adel Alawwadh, Osamah Barasheed, Mohammad Garnan, Sarah McIntyre, Harunor Rashid, Nadia Badawi, Gulam Khandaker

**Affiliations:** 1 Public Health Authority (PHA), 13351 Riyadh, Saudi Arabia; 2 The Children’s Hospital at Westmead Clinical School, Faculty of Medicine and Health, The University of Sydney, Westmead, NSW 2145 Australia; 3 Public Health Agency, Ministry of Health, 12382 Riyadh, Saudi Arabia; 4 King Fahad Center, Children with Disability Association (CDA), 12273 Riyadh, Saudi Arabia; 5 Khamis Mushayt Maternity and Children Hospital (KMMCH), 62454 Khamis Mushayt, Saudi Arabia; 6 The Executive Administration of Research and Innovation, King Abdullah Medical City in Holy Capital (KAMC-HC), 24246 Makkah, Saudi Arabia; 7 Public Health Administration, Asir Health, 62523 Abha, Saudi Arabia; 8 Cerebral Palsy Alliance Research Institute, Specialty of Child and Adolescent Health, Sydney Medical School, Faculty of Medicine and Health, The University of Sydney, Camperdown, NSW 2050 Australia; 9 Sydney Institute for Infectious Diseases, Faculty of Medicine and Health, The University of Sydney, Westmead, NSW 2145 Australia; 10 Grace Centre for Newborn Intensive Care, Sydney Children’s Hospital Network (SCHN), Westmead, NSW 2145 Australia; 11 Central Queensland Public Health Unit, Central Queensland Hospital and Health Service, Rockhampton, QLD 4700 Australia; 12 School of Health, Medical and Applied Sciences, Central Queensland University, Rockhampton, QLD 4701 Australia

**Keywords:** Cerebral palsy, Epidemiology, Pediatrics, Prevention, Register, Surveillance, Saudi Arabia

## Abstract

This protocol presents a comprehensive proposal for the establishment of the Saudi Cerebral Palsy Register (SCPR), a crucial project for investigating and addressing the prevalence, etiology, and management of cerebral palsy (CP) in Saudi Arabia. The SCPR will not only provide a robust database for ongoing research and analysis but will also serve as a platform for investigating the causes of CP, implementing preventative strategies, and improving the quality of care and outcomes for people with CP and their families in Saudi Arabia. Detailed case definitions, inclusion/exclusion criteria, and data collection protocols are discussed to ensure the integrity and comparability of the data. The plan also outlines strategic funding, institutional and government endorsement, sustainability considerations, potential challenges and proposed solutions, and expected outcomes and impact. These include creating research and educational opportunities, fostering regional and international collaborations, and significantly contributing to CP prevention strategies. Overcoming anticipated obstacles, such as stigma, institutional policies, and collaborations, and securing both necessary funding and endorsements are highlighted as critical for the success of the SCPR. The project is not only aligned with promote prevention of health risks, a target of Vision 2030 in Saudi Arabia, but is also expected to have a substantial impact on the health and quality of life of people with CP and their families in Saudi Arabia, serving as inspiration for similar efforts worldwide.

**Supplementary Information:**

The online version contains supplementary material available at 10.1007/s44197-024-00198-5.

## Introduction and Background

Cerebral palsy (CP) is defined as a group of disorders that occur in early life due to an injury to the growing brain. These disorders are neurodevelopmental, heterogeneous, and non-progressive [1]. Recently, estimates of the prevalence of CP ranging from 1 to nearly 4 per 1000 live births or per 1000 children have been reported worldwide. Most of these studies are predominantly from high-income countries (HICs), with only a few from low- and middle-income countries (LMICs) [2–4]. Interestingly, despite Saudi Arabia being a HIC, reliable CP prevalence data is lacking. Notably, the prevalence in HICs, specifically in Australia and Europe, was estimated at 1.5 per 1000, while in LMICs, it was considerably higher, at least double, ranging from 3 to 4 per 1000 [4]. However, there is still an urgent need for more studies to estimate the prevalence and trends of CP in Arab countries, including Saudi Arabia [5]. Saudi Arabia, a prosperous nation with a well-funded health system, is uniquely positioned to address these challenges. Its efforts and advancements in the healthcare sector can inspire other countries in the region. Intellectual disability, epilepsy, and feeding difficulties leading to malnutrition, pain, visual impairment, and hearing impairment are the most common co-occurring conditions in CP [6].

Over the last three decades, Australian and European CP registers have generated original data on the incidence, prevalence, trends, etiologies, and risk factors for CP in HICs. Australia pioneered the development of population-based CP registers. In 2007, the Australian Cerebral Palsy Register (ACPR), a nationwide CP register, was initiated. As part of this collaboration, de-identified data is contributed from all seven Australian state/territory CP registries to one common database. The Global Low- and Middle-Income Country Cerebral Palsy Register (GLM CPR) is a multi-country network of established and emerging CP registers in LMICs. The CP registers affiliated with the GLM CPR collect and collate information about the epidemiology of CP in LMICs using a standard harmonized protocol. Since its establishment in 2018, this network has grown exponentially. Currently, 11 LMIC-based CP registers are affiliated with GLM CPR, and the platform has ongoing collaborations to expand the network in future [7]. In 1998, the Surveillance of Cerebral Palsy in Europe (SCPE) was established, combining CP registers and surveys from nine European countries to form a framework for collaborative research [8–10], which has now grown to 31 members in 23 countries [11]. Considering Saudi Arabia's classification as a HIC [12], there is no national CP register that can be used for studies on prevalence, severity, etiology, associated impairments, and risk factors in Saudi Arabia, such as the aforementioned registers.

In Saudi Arabia, there is noticeable variation in the reported incidence and prevalence of neurological disorders, predominantly CP. Recent findings indicate that 6.9 out of every 1000 children experience significant chronic neurological disorders. Intellectual disability (ID) and CP are the most prevalent, with rates of 2.7/1000 and 2.3/1000, respectively, marking them as primary neurological disorders and the most frequent pediatric chronic conditions [13]. A systematic review highlighted an epilepsy prevalence of 6.5/1000, with the majority of these cases also having CP and/or intellectual disability [14]. While there is reference to a study that provides statistics on the incidence and prevalence of CP and childhood disability at the national level, a comprehensive, uniform dataset is conspicuously absent. Most existing studies are based on clinical presentation, emphasizing clinical rather than public health perspectives [15–17]. Most of these studies were conducted in specialized hospitals or centers, suggesting a potential bias toward more severe or complicated cases and a probable underestimation.

In addition to the clinical impact of CP, it is essential to recognize its broader social and psychological effects on families. While research indicates that caring for a child with CP can introduce challenges such as increased stress and a potential decrease in caregivers' quality of life, it is not universally dire for all. Factors contributing to these challenges include child behavior and cognitive difficulties, low self-efficacy of caregivers, and a lack of social support [18]. Caregivers might face personal difficulties, such as loss of employment, impaired concentration at work, disruption of family joy, and financial stress [19]. The lack of public knowledge regarding CP, its causes, and available treatment options can intensify this burden [20]. As children grow, the level of distress experienced by parents can be proportional to their motor and cognitive abilities and behavioral difficulties [21]. The perceived quality of life of people with CP can be influenced by various factors including the child's age, location of residence, and parents' education level [22]. By better understanding the population, families and children can be better supported to lead fulfilling lives. Therefore, broadening the objectives of a comprehensive database such as the proposed Saudi CP register is crucial. It should not only track clinical data, but also investigate, understand, and address these familial impacts. A national register in Saudi Arabia is essential to meet the needs of individuals with CP and their caregivers.

Under Vision 2030, Saudi Arabia's Health Sector Transformation Program (HSTP) seeks to overhaul the health sector into an integrated, effective, and comprehensive system that prioritizes the health of citizens, residents, and visitors. Grounded in the principle of value-based care, the program emphasizes transparency, financial sustainability, and a shift toward a prevention-focused model of care. A significant milestone in this transformation was the establishment of the Public Health Authority (PHA), formerly known as the National Center for Disease Prevention and Control. The PHA, as the proposed institution to oversee and manage the SCPR, is dedicated to reducing the incidence of both communicable and non-communicable diseases, overseeing their monitoring, and bolstering health through research on disease prevention and control [23, 24].

This protocol underscores the need for a CP register in Saudi Arabia, termed 'SCPR'. We outline its roadmap and key milestones, aligning with the broader objectives of the HSTP. This program, integral to Saudi Vision 2030, encompasses all health entities within Saudi Arabia, directing them toward key objectives built on the foundational pillars of Vision. The program's initiatives aim to enhance access to health services, elevate the quality and efficiency of care, and amplify prevention against health risks, underscoring the importance of public health and prevention services in the Vision 2030 framework.

## Methods and Technical Proposal for the SCPR

### Objectives and Strategic Goals

The primary focus of the SCPR is to collect current and accurate information on the causes, severity, and rehabilitation status of people with CP in Saudi Arabia. To achieve this, robust infrastructure for data collection is essential. Establishing such infrastructure not only ensures the accuracy and comprehensiveness of the data but also presents opportunities for job creation, further contributing to the local economy. Another strategic goal is to design a sustainable model for the SCPR, ensuring a comprehensive and continuous data collection method that includes the impact on families.

### Specific Objectives

- Continuously assess the prevalence and characteristics of CP in Saudi Arabia, including their effect on families. This data will provide valuable information on the extent of conditions within the population and broader family impacts.
- To expand our understanding of the causes and risk factors specific to CP in Saudi Arabia, shedding light on potential preventive measures, such as improving maternal health practices, and understanding other environmental or genetic factors prevalent in the region.
- To provide a foundation for future research, use the SCPR as a sampling framework to evaluate cost-effective intervention strategies. These strategies aim to improve functional rehabilitation, minimize associated or secondary impairments, and better manage the familial impact.
- To identify and quantify practice variations and patterns of care in clinical settings for patients with similar characteristics in a way that facilitates quality improvement.
- Advocate the creation of job opportunities at the government level specifically tailored for individuals with CP, leveraging the data and insights from the SCPR to highlight the potential and capabilities of this demographic.

### Ethical Considerations

The establishment and operation of the SCPR involves numerous ethical considerations that must be addressed to ensure the protection of participant rights, privacy, and data integrity.

- *Informed consent:* The SCPR must ensure that informed consent is obtained from all participants or their caregivers before inclusion in the register. This process involves a comprehensive explanation of the purpose of the register, the type of data collected, how the data will be used, stored, and shared, and the potential risks and benefits of participation. Participants will be assured of their right to withdraw from the register at any time without any repercussions (see Online Appendices 1 and 2, draft Participant Information Sheet, and draft Consent Form).
- *Privacy and Confidentiality:* Strict measures will be put in place to protect the privacy and confidentiality of all participants. Personal identifiers will be securely stored separately from another data to ensure confidentiality and will only be used for approved follow-up research, with the consent of the participant. Strict protocols for data access, storage, and transmission will be developed to prevent unauthorized access or data breaches.
- *Data Use and Sharing:* SCPR should establish clear policies on how collected data can be used and shared. This includes provisions for sharing de-identified data with other researchers or institutions and the conditions under which such sharing can occur. Participants must be informed of these policies, and their consent must be obtained. To safeguard against cybercrimes and data breaches, standard operating procedures (SOPs) will be implemented for all staff dealing with data. Necessary measures will be implemented to minimize the vulnerabilities of all hardware and software. A detailed SOPs for data protection and cybersecurity measures will be developed.
- *Protection of Vulnerable Populations:* Given that the SCPR will involve children, people with CP, and families with financial difficulties, who are considered vulnerable populations, special protections will be in place. This includes ensuring that the benefits of participation outweigh any potential risks and that the well-being of the individual with CP and the family is always prioritized. Consider how families may benefit from participation and how to protect them, perhaps by connecting them with additional resources or support services. Regular audits and evaluations should be conducted to ensure compliance with the ethical guidelines and standards.
- *Transparency and Accountability:* The SCPR should commit to transparency and accountability for all operations. This includes regular reporting on the activities, findings, and impacts of the register and responding to any complaints or concerns raised by participants or other stakeholders.
- *Advisory Group:* To ensure that the SCPR remains aligned with the needs and concerns of its participants and other key stakeholders, an Advisory Group will be established. This group comprises key stakeholders, including families, healthcare professionals, and other relevant experts. The Advisory Group will provide guidance on the ethical considerations mentioned above, ensuring that the perspectives of those directly impacted by the register are always considered. They will also review and provide feedback on SOPs, data use policies, and other operational aspects of the SCPR.

### Setting

Initially, SCPR is proposed to be established with the collaboration of the Children with Disability Association (CDA) in Riyadh as a primary starting point. This is because a significant proportion of people with CP benefit from its two centers in Riyadh, thus offering a robust initial sampling frame for the SCPR. Furthermore, PHA in Riyadh is envisioned as the primary institution that owns and manages SCPR. This aligns with PHA's mandate for health data management and surveillance of health data in Saudi Arabia. A Memorandum of Understanding (MOU) will be established between PHA and CDA if needed.

The choice of the main centers for the initial stage of the SCPR provides several advantages. First, it allows for comprehensive and concentrated collection of high-quality data from a diverse and representative sample of people with CP. Second, it enables the SCPR team to establish strong partnerships with healthcare providers at these centers, fostering a collaborative environment for successful implementation.

Furthermore, by initially focusing on the main centers, the SCPR can optimize the development and refinement of its methodologies, data collection protocols, and operational procedures within a controlled setting. This approach also ensures the efficient use of resources during the register's early stages, and allows for a thorough evaluation and review process to identify and address areas of improvement.

To manage the ownership of the SCPR, conversations with healthcare leaders and key stakeholders will be engaged, including the advisory group. As previously mentioned, this group will comprise key stakeholders, including families, healthcare professionals, and other relevant experts, ensuring that the perspectives of those directly impacted by the register are always considered. Understanding the intricacies of the health data ecosystem, especially in the context of ongoing healthcare transformations, is crucial. Collaborative ownership and financial support can potentially be sought from relevant government entities, non-profit organizations, or private sector partners interested in improving the care of patients with CP and their families.

Once the SCPR has been established, evaluated, and refined within specific centers, the plan should be to gradually expand the register to include both community- and hospital-based services throughout Saudi Arabia. This phased expansion approach will ensure that the SCPR maintains high standards of data quality, integrity, and ethics as it grows, ultimately leading to a comprehensive national CP register that covers all of Saudi Arabia.

The initial setting of the SCPR within specific rehabilitation centers in Saudi Arabia lays a solid foundation for the development of the register, with an expanded collaborative framework including the ‘Authority for People with Disabilities (APD) and the King Salman Center for Disability Research (KSCDR)’. This approach provides a pathway for its eventual expansion to encompass both community- and hospital-based services, leveraging the expertise and reach of these additional key stakeholders, ensuring comprehensive coverage on a national scale.

### Case Definition and Ascertainment

CP is defined as *“a group of permanent disorders of the development of movement and posture, causing activity limitation, that are attributed to non-progressive disturbances that occurred in the developing fetal or infant brain. The motor disorders of cerebral palsy are often accompanied by disturbances of sensation, perception, cognition, communication, and behavior, by epilepsy, and secondary musculoskeletal problems”* [25]. It is characterized by the following criteria [26, 27]:

- CP is a comprehensive term that represents various disorders.
- It is a permanent condition; however, its manifestation is not static and can change over time.
- The disorder manifests itself as movement and/or postural impairment that affects motor function.
- The root cause of the disorder is non-progressive interference, lesions, or abnormalities.
- Interference, lesions, or abnormalities originate in the brain during immature developmental stages.

This definition provides a clear and consistent standard for the identification and classification of CP cases, ensuring the integrity and comparability of data collected by the SCPR.

### Inclusion/Exclusion Criteria

The following criteria ensure that the SCPR will collect data from the target population of people with CP in Saudi Arabia, and will not include data from individuals with other conditions that could potentially confound the results.

Inclusion criteria:

- The diagnosis of CP as defined in the previous section.
- Individuals of all ages at the time of recruitment, with a confirmed diagnosis of CP by the age of 5 years, to ensure comprehensive data on CP in Saudi Arabia.
- Living in Saudi Arabia, to ensure that the data collected is representative of the population the SCPR aims to serve.

Exclusion criteria:

- Non-residents or temporary visitors to Saudi Arabia (e.g., those who only come for Hajj, Umrah, or tourism).
- Individuals with other neurological disorders that do not meet the criteria for a CP diagnosis.
- Individuals who did not reach the age of 5 and did not have a confirmed diagnosis of CP.
- Children who were diagnosed with CP and passed away before the age of 2 years.

### Data Collection and Handling

Data handling and management are integral for maintaining the integrity and ethical standards of any register. For the SCPR, the following principles must be upheld.

- *Coding:* All data collected, managed, and analyzed will be coded. Unique identification codes will be assigned to each participant. This will maintain the privacy and confidentiality of the individuals included in the register for the purpose of research and publications, while retaining personal information for contact and follow-up purposes.
- *Access Control:* Data access must be strictly regulated. Only authorized persons will be granted permission to view and use the dataset. Separate ethics approval will be necessary for projects seeking to use the data, and all provided data will be de-identified to further safeguard the privacy of the participants.
- *Confidentiality:* All reports, presentations, and academic articles derived from SCPR should not contain identifiable information. This ensures that no personal details can be traced back to the individuals involved in the study, maintaining their privacy.
- *Data Ownership:* The PHA in Riyadh is the proposed institution to own and manage the SCPR, aligning with its mandate for health data management and surveillance of health data. They have the responsibility for overseeing data storage, access, and usage, ensuring that all procedures align with the ethical guidelines and standards for data management in Saudi Arabia.
- *Data Storage:* Data collected must be securely stored in an encrypted database. Regular backup of the database should be performed to prevent data loss.
- *Data Monitoring and Audit:* Regular data monitoring and auditing must be carried out to ensure data quality and consistency. Any inconsistencies or issues identified during these checks must be promptly corrected.

### Data Variables

The SCPR's core variable list, detailed in Online Appendix 3, has been meticulously developed in consultation with the ACPR and GLM CPR groups to ensure comprehensive and relevant data collection. This list includes metabolic and genetic factors as key etiological variables or mimickers of CP, particularly pertinent in consanguineous marriage among populations such as in Saudi Arabia. It encompasses the demographic details of the person with CP, their parents, and the health professionals involved in their care. In addition to essential clinical information including birth details, diagnosis, severity, associated impairments, the SCPR captures detailed metabolic and genetic information, aligned with the findings in the field [13, 28, 29].

The list of variables emphasizes the importance of ethical considerations. For instance, informed consent is paramount and participants' privacy is maintained by coding all data and using unique identification codes.

### Data Analysis

Data analysis for the SCPR should be an ongoing and regular iterative process. The analysis approach will be primarily quantitative, focusing on descriptive statistics to provide a comprehensive overview of the prevalence, severity, and etiology of CP in Saudi Arabia. This can include frequency counts, birth prevalence estimates, central tendency (mean and median), and measures of dispersion (range and standard deviation). To understand the evolution and potential shifts in the landscape of CP in Saudi Arabia, it is essential to analyze trends over time. This longitudinal analysis will help identify patterns, changes in prevalence rates, and any emerging risk factors or patterns in the data over successive years. Such trend analyses are crucial for policymaking, resource allocation, and targeted intervention design. Additional statistical analyses can be performed to explore the potential associations between risk factors and the occurrence of CP. These analyses include chi-square tests for categorical variables, *t*-tests for comparing means, and regression analyses to examine the relationships between multiple variables. Analyses will be conducted using suitable statistical software, and the choice of software will be determined by the specific requirements of the analysis. All findings should be reported regularly (e.g., annual reports and scientific journals).

## Implementation of Proposed SCPR

### Feasibility

The implementation of the SCPR requires careful planning and feasibility assessment, considering several vital factors. Resource availability, including personnel and technological infrastructure, is an essential consideration that requires in-depth evaluation of the existing resources necessary for effective data collection, management, and analysis. To ensure accurate and consistent data collection across all participating centers, it is equally important to invest in staff training and capacity-building. The participation of stakeholders, from healthcare providers to patients and their families, and relevant governmental and non-governmental organizations, is critical to gaining wide-ranging support for SCPR. Recognizing the importance of diverse stakeholder inputs, we mentioned the establishment of an Advisory Group earlier. This will provide strategic direction, ensuring that the perspectives of those directly impacted by the register are always considered. They will also review and provide feedback on the SOPs, data use policies, and other operational aspects of the SCPR, as detailed in the Ethical Considerations section. Before a phased national rollout, it would be prudent to conduct pilot tests in one or two centers to identify potential challenges and rectify them prior to full-scale implementation. This approach aligns with the previously mentioned strategy of initially establishing the SCPR within the main rehabilitation centers.

### Branding and Communication Strategy

Branding plays a pivotal role in making a project identifiable and memorable. The objective of the SCPR is to design a distinctive and easily recognizable logo that not only captures the essence of the mission, improving the understanding and management of CP in Saudi Arabia, but is also culturally sensitive and resonates with the identity of Saudi Arabia. This logo will serve as the visual identity of the SCPR and will be prominently featured in all publicly available documents/resources related to the project.

In addition to the logo, a dedicated website for the SCPR should be established. This website will serve as a central hub for information related to the SCPR, offering the latest updates, research findings, upcoming events, and resources tailored to healthcare providers, researchers, and families affected by CP. The website will be designed to be user-friendly and accessible, ensuring that the information is easily retrievable and comprehensible. Moreover, with the advancement of artificial intelligence (AI) and data analytics, websites will incorporate powerful analytics software to generate interactive real-time data, enhancing user engagement and understanding.

To ensure broad outreach and participation, the potential use of social media platforms should be considered. However, the selection of platforms and the nature of content dissemination will be deliberated in consultation with the advisory committee, ensuring alignment with the policies in Saudi Arabia and the best practices in healthcare communication. In addition, a clear policy or set of SOPs will be established to guide the use of social media, ensuring that privacy, confidentiality, and content quality are maintained.

The overarching aim is for the SCPR to be more than a register. It should serve as a platform to foster connections, share knowledge, and ignite collaboration. To further this goal and ensure that the perspectives of those directly affected by CP are always at the forefront, the establishment of a consumer advisory group will be considered. This group will play a crucial role in guiding the SCPR's communication and outreach strategies, ensuring that they are aligned with the needs and concerns of the CP community in Saudi Arabia.

### Funding and Sustainability

The financial considerations for establishing and maintaining a comprehensive register, such as the SCPR, are significant. To ensure that the register can effectively and continuously contribute valuable data to the study and management of CP in Saudi Arabia, it is critical to secure sustainable funding. Potential funding sources include government bodies, non-profit organizations, and research grants. We recommend an initial search for feasibility funding as part of a strategic funding approach. This would allow the SCPR to demonstrate its potential impact and establish preliminary achievements, which can then be used to leverage more substantial grants. To manage this process effectively, it would be beneficial to establish a dedicated team responsible for identifying potential funding opportunities and preparing robust grant applications. This team would highlight the impact and potential benefits for the health and well-being of people with CP in Saudi Arabia, thus improving the chances of securing necessary funds.

### Challenges and Possible Solutions

The implementation of the SCPR may face several challenges that need to be addressed proactively. The stigma associated with CP and other disabilities can pose challenges to data collection and participation. Cultural sensitivities and strategies to address any potential stigma, such as involving community leaders in awareness and advocacy campaigns, should be considered. Additionally, institutional policies and collaborations can present challenges. Coordination and collaboration between different governmental and non-governmental institutions are crucial to the success of the register. Establishing clear policies, agreements, and an understanding among these institutions will facilitate smoother operations. Despite these potential obstacles, the SCPR is expected to be a significant milestone in understanding and managing CP in Saudi Arabia. A systematic approach to identifying and addressing these challenges will ensure the sustainability and success of the SCPR.

## Expected Outcomes and Implications

The establishment of the SCPR presents a multitude of opportunities and potential positive outcomes, particularly in the areas of research, education, and international collaboration.

### Research and Educational Opportunities

The SCPR will provide a substantial and invaluable database of CP in Saudi Arabia. This will not only enable a comprehensive understanding of the prevalence, etiology, and risk factors of CP but also provide a solid platform for future research. The rich data from the SCPR can be used for epidemiological studies, clinical trials, and evaluation of the effectiveness of various interventions.

In terms of educational opportunities, the SCPR opens avenues for collaborations with international academic and professional bodies, including, but not limited to, the American Academy of Cerebral Palsy and Developmental Medicine (AACPDM), the Australasian Academy of Cerebral Palsy and Developmental Medicine (AusACPDM), and the European Academy of Childhood Disability (EACD). These collaborations can foster the exchange of knowledge, skills, and best practices, which will be instrumental in improving the standards of care and rehabilitation for individuals with CP and their families in Saudi Arabia.

### Regional and International Collaborations

The SCPR has the potential to serve as a beacon for regional collaboration, aligning with similar projects such as the existing CP Register in Jordan (CPUP- Jordan) and prospective registers such as the CP register in Kuwait [30, 31]. Beyond regional collaboration, there is a vast horizon for international partnerships. These include ties with the ACPR, SCPE, New Zealand Cerebral Palsy Register (NZCPR), Bangladesh Cerebral Palsy Register (BCPR), Sri Lankan Cerebral Palsy Register (SLCPR), Global LMIC Cerebral Palsy Register (GLM CPR), and other registers. These international collaborations can pave the way for sharing data, methodologies, and experiences, enriching the global understanding of CP. Given the regional significance and the shared cultural and health dynamics, there's also a compelling case for the establishment of a Gulf Cooperation Council (GCC) CP Register. Such a regional register would further consolidate data, resources, and expertise, thus amplifying the impact of individual national registers.

### Contribution to CP Prevention

In the long term, SCPR is expected to contribute significantly to the prevention of CP and reduce its severity. By identifying the risk factors and understanding the etiology of CP in the Saudi context, it is possible to design and implement effective prevention strategies. Furthermore, the SCPR data can be used to advocate appropriate public health policies and interventions that address the specific needs of the population with CP in Saudi Arabia. Ultimately, the SCPR is poised to play a pivotal role in improving the quality of life of individuals with CP and their families.

## Conclusion and Recommendation

The establishment of the SCPR is a crucial step toward understanding and addressing CP in Saudi Arabia. SCPR will not only provide valuable information on the prevalence, etiology, and severity of CP in Saudi Arabia but will also pave the way for comprehensive and cost-effective intervention strategies. This is also in line with the scientific recommendations for such registers [32]. The expected results, including enhanced research and educational opportunities, and the promotion of regional and international collaborations, underscore the potential impact of the SCPR. However, its success depends on the ability to overcome anticipated challenges, such as stigma, lack of research infrastructure, and the need for institutional policies and collaborations. Therefore, it is recommended to secure the necessary funding, foster a supportive environment at all levels, and maintain a patient-centered approach while addressing these challenges. Furthermore, continuous evaluation and improvement of the SCPR will be vital to ensure its sustainability and adaptability to the evolving needs of people with CP and their families in Saudi Arabia. The proposed SCPR has the potential to significantly contribute to the improvement of the health and quality of life of people with CP and their families in Saudi Arabia. It is hoped that this project will inspire similar efforts in the region and contribute to a global understanding of CP, its causes, and effective interventions.

### Supplementary Information

Below is the link to the electronic supplementary material.

Supplementary file1 (DOCX 28 KB)

Supplementary file2 (DOCX 33 KB)

Supplementary file3 (DOCX 33 KB)

## Data Availability

Not applicable.

## Abbreviations

AACPDM: American Academy of Cerebral Palsy and Developmental Medicine
ACPR: Australian Cerebral Palsy Register
AI: Artificial intelligence
AusACPDM: Australasian Academy of Cerebral Palsy and Developmental Medicine
APD: Authority for People with Disabilities
BCPR: Bangladesh Cerebral Palsy Register
CDA: Children with Disability Association
CP: Cerebral palsy
CPUP-Jordan: Cerebral Palsy Follow-up Registry in Jordan
EACD: European Academy of Childhood Disability
GCC: Gulf Cooperation Council
GLM CPR: Global Low- and Middle-Income Country Cerebral Palsy Register
HICs: High-income countries
HSTP: Health Sector Transformation Program
ID: Intellectual disability
KSCDR: King Salman Center for Disability Research
LMICs: Low–middle-income countries
MOU: Memorandum of understanding
NZCPR: New Zealand Cerebral Palsy Register
PHA: Public Health Authority
SCPE: Surveillance of Cerebral Palsy in Europe
SCPR: Saudi Cerebral Palsy Register
SLCPR: Sri Lankan Cerebral Palsy Register
SOPs: Standard operating procedures

## References

[1] Vitrikas K, Dalton H, Breish D (2020). Cerebral palsy: an overview. Am Fam Physician.

[2] Jahan I, Muhit M, Hardianto D, Laryea F, Chhetri AB, Smithers-Sheedy H (2021). Epidemiology of cerebral palsy in low- and middle-income countries: preliminary findings from an international multi-centre cerebral palsy register. Dev Med Child Neurol.

[3] Khandaker G, Smithers-Sheedy H, Islam J, Alam M, Jung J, Novak I (2015). Bangladesh Cerebral Palsy Register (BCPR): a pilot study to develop a national cerebral palsy (CP) register with surveillance of children for CP. BMC Neurol.

[4] McIntyre S, Goldsmith S, Webb A, Ehlinger V, Hollung SJ, McConnell K (2022). Global prevalence of cerebral palsy: a systematic analysis. Dev Med Child Neurol.

[5] Mushta SM, King C, Goldsmith S, Smithers-Sheedy H, Badahdah AM, Rashid H (2022). Epidemiology of cerebral palsy among children and adolescents in arabic-speaking countries: a systematic review and meta-analysis. Brain Sci.

[6] Jan MM (2006). Cerebral palsy: comprehensive review and update. Ann Saudi Med.

[7] CP Register Australia. Australian cerebral palsy register report 2023. 2023.

[8] Cans C (2007). Surveillance of cerebral palsy in Europe: a collaboration of cerebral palsy surveys and registers. Dev Med Child Neurol.

[9] Cans C, Surman G, McManus V, Coghlan D, Hensey O, Johnson A (2004). Cerebral palsy registries. Semin Pediatr Neurol.

[10] Goldsmith S, Garcia Jalon G, Badawi N, Blair E, Garne E, Gibson C (2018). Comprehensive investigation of congenital anomalies in cerebral palsy: protocol for a European–Australian population-based data linkage study (The Comprehensive CA-CP Study). BMJ Open.

[11] Gokina NI, Fairchild RI, Prakash K, DeLance NM, Bonney EA (2021). Deficiency in CD4 T cells leads to enhanced postpartum internal carotid artery vasoconstriction in mice: the role of nitric oxide. Front Physiol.

[12] The World Bank. World bank country and lending groups. The World Bank. 2023. https://datahelpdesk.worldbank.org/knowledgebase/articles/906519-world-bank-country-and-lending-groups. Accessed 16 May 2023

[13] Al Salloum AA, El Mouzan MI, Al Omar AA, Al Herbish AS, Qurashi MM (2011). The prevalence of neurological disorders in Saudi children: a community-based study. J Child Neurol.

[14] Benamer HT, Grosset DG (2009). A systematic review of the epidemiology of epilepsy in Arab countries. Epilepsia.

[15] Al-Asmari A, Al Moutaery K, Akhdar F, Al JM (2006). Cerebral palsy: incidence and clinical features in Saudi Arabia. Disabil Rehabil.

[16] Al-Rajeh S, Bademosi O, Awada A, Ismail H, Al-Freihi H, Dawodu A (1995). Community survey of neurological disorders in Saudi Arabia: Results of the pilot study in Agrabiah. Ann Saudi Med.

[17] Al-Sulaiman AA (2001). Epilepsy in Saudi children with cerebral palsy. Saudi Med J.

[18] Pousada M, Guillamón N, Hernández-Encuentra E, Muñoz E, Redolar D, Boixadós M (2013). Impact of caring for a child with cerebral palsy on the quality of life of parents: a systematic review of the literature. J Dev Phys Disabil.

[19] Henry G, Webb A, Galea C, Pearce A, Balde I, Garrity F (2023). Out-of-pocket costs for families and people living with cerebral palsy in Australia. PLoS ONE.

[20] Olawale OA, Deih AN, Yaadar RK (2013). Psychological impact of cerebral palsy on families: the African perspective. J Neurosci Rural Pract.

[21] Majnemer A, Shevell M, Law M, Poulin C, Rosenbaum P (2012). Indicators of distress in families of children with cerebral palsy. Disabil Rehabil.

[22] Koltuniuk A, Rozensztrauch A, Budzinska P, Rosinczuk J (2019). The quality of life of polish children with cerebral palsy and the impact of the disease on the family functioning. J Pediatr Nurs.

[23] Health Sector Transformation Program. Health sector transformation program delivery plan: vision 2030, Kingdom of Saudi Arabia. 2021. https://www.vision2030.gov.sa/v2030/vrps/hstp/. Accessed 16 May 2023

[24] Alasiri AA, Mohammed V (2022). Healthcare transformation in Saudi Arabia: an overview since the launch of vision 2030. Health Serv Insights.

[25] Rosenbaum P, Paneth N, Leviton A, Goldstein M, Bax M, Damiano D (2007). A report: the definition and classification of cerebral palsy April 2006. Dev Med Child Neurol Suppl.

[26] Gulati S, Sondhi V (2018). Cerebral palsy: an overview. Indian J Pediatr.

[27] Patel DR, Neelakantan M, Pandher K, Merrick J (2020). Cerebral palsy in children: a clinical overview. Transl Pediatr.

[28] Hakami WS, Hundallah KJ, Tabarki BM (2019). Metabolic and genetic disorders mimicking cerebral palsy. Neurosci J.

[29] Smithers-Sheedy H, Badawi N, Blair E, Cans C, Himmelmann K, Krägeloh-Mann I (2014). What constitutes cerebral palsy in the twenty-first century?. Dev Med Child Neurol.

[30] Almutairi AB, AlAbdullkarim AE, Al-Shatti AA (2023). Establishing a cerebral palsy registry in Kuwait: an exploratory study. J Taibah Univ Med Sci.

[31] Almasri NA, Saleh M, Abu-Dahab S, Malkawi SH, Nordmark E (2018). Development of a cerebral palsy follow-up registry in Jordan (CPUP-Jordan). Child Care Health Dev.

[32] McIntyre S, Novak I, Cusick A (2010). Consensus research priorities for cerebral palsy: a Delphi survey of consumers, researchers, and clinicians. Dev Med Child Neurol.

