# Supplementary material for: Saudi Cerebral Palsy Register (SCPR): Protocol on the Methods and Technical Details
Source: J Epidemiol Glob Health. 2024 Feb 15;14(2):453–61. doi: 10.1007/s44197-024-00198-5 (PMC11176122; doi:10.1007/s44197-024-00198-5)
Supplement: Supplementary file 1 — Supplementary file1 (DOCX 28 KB) [file 44197_2024_198_MOESM1_ESM.docx]

| أنا، [ الاسم الرباعي ] | I, [ Full Name ] |
| --- | --- |
|  |  |
| أوافق على تسجيل | herby give consent to the inclusion of |
|  |  |
| [ الاسم الرباعي ] | [ Full Name ] |
|  |  |
| في السجل السعودي للشلل الدماغي، بصفتي: | on the Saudi Cerebral Palsy Register, being: |
|  |  |
| ( نفس الشخص \| ولي الأمر \| الشخص المسؤول ) | ( myself \| parent \| person responsible ) |
|  |  |
| **(يرجى وضع دائرة حول الإجابة المناسبة أعلاه وفي جميع أنحاء هذا المستند).** | **(Please circle the appropriate response above and throughout this document).** |
|  |  |
| لقد قرأت وفهمت ورقة المعلومات وتمت الإجابة على أسئلتي بطريقة مرضية. أفهم أنه قد لا تكون هناك فائدة مباشرة من عضويتي في هذا السجل، وأنه لن يتم الاستفادة مادياً مقابل الانضمام إلى السجل. أدرك أنه يجب علي الاحتفاظ بنسخة من نموذج الموافقة بعد استكماله ، ونسخة من ورقة المعلومات للمشاركين والأسر/ مقدمي الرعاية. | I have read and understood the information sheet and had any questions answered to my satisfaction. I understand that an individual may not directly benefit from membership of the SCPR and that no payment will be made for joining the register. I am aware that I should retain a copy of the consent form, when completed, and the information sheet for my records. |
|  |  |
| **أوافق على:** | **I consent to:** |

| نعم | لا | جمع وتسجيل وتخزين دائم للمعلومات المتعلقة (بي / بطفلي / الشخص) في السجل. قد يتضمن ذلك الرجوع إلى تفاصيل الولادة والسجلات الطبية. | The collection, recording and permanent storage of information relating to (me / my child / the person), on the SCPR. This may involve consulting birth and medical records. | No | Yes |
| --- | --- | --- | --- | --- | --- |
|  |  |  |  |  |  |
| نعم | لا | نقل المعلومات مجهولة الهوية إلى السجل السعودي للشلل الدماغي. | Transfer of de-identified information to the Saudi Cerebral Palsy Register. | No | Yes |
|  |  |  |  |  |  |
| نعم | لا | تلقي الدعوات من وقت لآخر من موظفي السجل للمشاركة في الدراسات البحثية. | Receiving invitations from time to time from SCPR staff to participate in research studies. | No | Yes |
|  |  |  |  |  |  |
| نعم | لا | الممارسين الصحيين الذين رشحتهم (أنا / طفلي / الشخص) الذي سيتم الاتصال به للمساعدة في إكمال و / أو التحقق من التفاصيل الموجودة في السجل. يتم سرد هؤلاء الممارسين الصحيين في استمارة التسجيل. | Health professional/s nominated by (me / my child / the person) to be contacted to assist in completing and / or verifying the details on the register. These health professionals are listed on the registration form. | No | Yes |

| التوقيع |  | | | | | | | | | |  | | | | | | | | | | Signed |
| --- | --- | --- | --- | --- | --- | --- | --- | --- | --- | --- | --- | --- | --- | --- | --- | --- | --- | --- | --- | --- | --- |
|  |  | | | | | | | | | |  | | | | | | | | | |  |
| التاريخ |  |  | / |  |  | / |  |  |  |  |  |  |  |  | / |  |  | / |  |  | Date |

| **يستخدم فقط إذا تمت المناقشة مع الممارس الصحي** | **Use only if discussed with a health professional** |
| --- | --- |
|  |  |
| أنا ، بصفتي ممارساً صحياً ، أشهد أنني أوضحت المشروع (للشخص نفسه / الوالد و / أو الشخص المسؤول) وقد تم فهم ما يترتب على التسجيل، وقد وافق / وافقت بكل حرية. | I, being a health professional certify that I have explained the project to the person / parent and / or person responsible and consider that he / she understands what is involved and has freely given his / her consent. |

| التوقيع |  | | | | | | | | | |  | | | | | | | | | | Signed |
| --- | --- | --- | --- | --- | --- | --- | --- | --- | --- | --- | --- | --- | --- | --- | --- | --- | --- | --- | --- | --- | --- |
|  |  | | | | | | | | | |  | | | | | | | | | |  |
| التاريخ |  |  | / |  |  | / |  |  |  |  |  |  |  |  | / |  |  | / |  |  | Date |
|  |  | | | | | | | | | |  | | | | | | | | | |  |
| الاسم |  | | | | | | | | | |  | | | | | | | | | | Name |
|  |  | | | | | | | | | |  | | | | | | | | | |  |
| المسمى الوظيفي |  | | | | | | | | | |  | | | | | | | | | | Title |
