# Supplementary material for: Saudi Cerebral Palsy Register (SCPR): Protocol on the Methods and Technical Details
Source: J Epidemiol Glob Health. 2024 Feb 15;14(2):453–61. doi: 10.1007/s44197-024-00198-5 (PMC11176122; doi:10.1007/s44197-024-00198-5)
Supplement: Supplementary file 2 — Supplementary file2 (DOCX 33 KB) [file 44197_2024_198_MOESM2_ESM.docx]

| **1** | Variables for demographic characteristics for person with CP | |
| --- | --- | --- |
|  | **Variable** | **Description** |
| 1.1 | Name of person with CP | *Full name* |
| 1.2 | Sex | *Male or female* |
| 1.3 | Date of birth | *Both in Hijri and Gregorian* |
| 1.4 | Place of birth | *As mentioned in Birth Certificate* |
| 1.5 | Address | *National address* |
| 1.6 | Phone | *Mobile phone preferred* |
| 1.7 | Email |  |
| 1.8 | Type of accommodation |  |
| 1.9 | Address at age 5 | *National address* |
| 1.10 | Immunisation status | *Up to date, not (reason if known)* |

| **2** | Variables for demographic characteristics for person responsible * | |
| --- | --- | --- |
|  | **Variable** | **Description** |
| 2.1 | Name | *Full name* |
| 2.2 | Sex | *Male or female* |
| 2.3 | Type of relationship | *To the person with CP* |
| 2.4 | Address | *National address* |
| 2.5 | Phone | *Mobile phone preferred* |
| 2.6 | Email |  |
| * For at least two persons (e.g., parents, one parent and one emergency contact, etc). | | |

| **3** | Variables for health professional * | |
| --- | --- | --- |
|  | **Variable** | **Description** |
| 3.1 | Name | *Full name* |
| 3.2 | Title | *Professional title* |
| 3.3 | Phone | *Work phone (and extension)* |
| 3.4 | Workplace |  |
| 3.5 | Address | *National address of the workplace* |
| 3.6 | Email | *Official work email* |
| 3.7 | Collaboration with other entities | *To be specified (e.g., KSCDR)* |
| * Preferred to have details of two health professional persons. | | |

| **4** | Variables for birth details of person with CP | |
| --- | --- | --- |
|  | **Variable** | **Description** |
| 4.1 | Birthplace | *Hospital name/centre name/home birth…* |
| 4.2 | If home birth | *Planned/unplanned. State the cause of home birth* |
| 4.3 | Birth weight | *In gram* |
| 4.4 | Gestational age | *In weeks* |
| 4.5 | Type of care | *Routine, NICU, nursery. State the length of stay in days* |
| 4.6 | MRI | *Yes, no. Where if yes* |
| 4.7 | Multiple birth? | *Yes, no. What type if yes* |
| 4.8 | Birth order | *1^st^, 2^nd^, …* |
| 4.9 | Assistance with conception? | *Yes, no. What type if yes (if known)* |
| 4.10 | Number of previous live births | *number* |
| 4.11 | Previous stillbirths (>20 weeks gestation) | *Number* |
| 4.12 | Previous miscarriages (<20 weeks gestation) | *Number* |
| 4.13 | Congenital anomaly? | *Yes, no. Specify if yes* |

| **5** | Variables for father’s details | |
| --- | --- | --- |
|  | **Variable** | **Description** |
| 5.1 | Name | *Full name* |
| 5.2 | Date of birth | *Both in Hijri and Gregorian* |
| 5.3 | Place of birth | *City name, country name* |
| 5.4 | Educational level at time of child’s birth |  |
| 5.5 | Occupation at time of child’s birth |  |

| **6** | Variables for mother’s details | |
| --- | --- | --- |
|  | **Variable** | **Description** |
| 6.1 | Name | *Full name* |
| 6.2 | Date of birth | *Both in Hijri and Gregorian* |
| 6.3 | Place of birth | *City name, country name* |
| 6.4 | Educational level at time of child’s birth |  |
| 6.5 | Occupation at time of child’s birth |  |

| **7** | Variables for parents’ consanguinity | |
| --- | --- | --- |
|  | **Variable** | **Description** |
| 7.1 | Consanguineous marriage? | *Yes, no. Type of consanguinity if yes* |

| **8** | Variables for family history of CP | |
| --- | --- | --- |
|  | **Variable** | **Description** |
| 8.1 | Positive family history? | *Yes, no. Provide details if yes* |
| 8.2 | Known Metabolic or Genetic Conditions in Family? | *Yes, no. Provide details if yes* |

| **9** | Variables for problems during pregnancy | |  |
| --- | --- | --- | --- |
|  | **Variable** | **Description** |  |
| 9.1 | Problems during pregnancy? | *Haemorrhage, pre-eclampsia/eclampsia, PMROM/premature labour, problems with placenta, maternal blood clotting, exposure to toxins or medications, maternal thyroid problems, Rh incompatibility, unknown, other/unspecified problem ** |  |
| * Details to be given in the ‘Comments’ variable. | | | |

| **10** | Variables for mode of delivery | |
| --- | --- | --- |
|  | **Variable** | **Description** |
| 10.1 | Mode of delivery? | *NSVD, C-S (planned/emergency), instrumental delivery* |

| **11** | Variables for clinical details of person with CP (to be left blank if unsure) | |
| --- | --- | --- |
|  | **Variable** | **Description** |
| 11.1 | Age at which CP was formally diagnosed | *Years, months* |
| 11.2 | Type of CP *  (Spasticity, dyskinesia, ataxia, hypotonia, resolved by age 5 **, known syndrome – not CP **, unknown syndrome – not CP **, unknown | *Spasticity includes (Lt hemiplegia/monoplegia, Rt hemiplegia/monoplegia, diplegia, triplegia, quadriplegia)*  *Dyskinesia includes (mainly athetosis, mainly dystonia)* |
| 11.3 | Treatment Approaches Used for CP | *Text Field - to capture the specific treatment modalities used* |
| 11.4 | Frequency of Interventions/Therapies | *Text Field - to record how often the patient receives various therapies or interventions* |
| * Each type has these three options corresponding to the description (main type at initial diagnosis, main type at or over age 5, secondary type at or over age 5).  ** These types have only the first two options corresponding to the description. | | |

| **12** | Variables for severity of CP according to GMFCS * | |
| --- | --- | --- |
|  | **Variable** | **Description** |
| 12.1 | Level I | *At initial diagnosis, at or over age 5* |
| 12.2 | Level II | *Same as above* |
| 12.3 | Level III | *Same as above* |
| 12.4 | Level IV | *Same as above* |
| 12.5 | Level V | *Same as above* |
| * GMFCS sheet to be provided to each participating centre. | | |

| **13** | Variables for ability to handle objects in daily life according to MACS * | |
| --- | --- | --- |
|  | **Variable** | **Description** |
| 13.1 | Level I | *At or over age 4* |
| 13.2 | Level II | *Same as above* |
| 13.3 | Level III | *Same as above* |
| 13.4 | Level IV | *Same as above* |
| 13.5 | Level V | *Same as above* |
| * MACS sheet to be provided to each participating centre. | | |

| **14** | Variables for birth defects and/or syndromes | |
| --- | --- | --- |
|  | **Variable** | **Description** |
| 14.1 | Birth defect? | *Yes, no. Provide details if yes* |
| 14.2 | Known syndrome? | *Yes, no. Provide details if yes* |
| 14.3 | Indication of Metabolic or Genetic Syndromes? | *Yes, no. Provide details if yes* |
| 14.4 | Genetic testing conducted? | *Yes (Microarray, Whole Exome, Whole Genome, Other – provide details, type of testing unknown), no, unknown* |

| **15** | Variables for presence of associated impairments | |
| --- | --- | --- |
|  | **Variable** | **Description** |
| 15.1 | Epilepsy? | *Yes, no, resolves by age 5, unknown* |
| 15.2 | Intellectual? | *No, probably no, probably some, mild, moderate, severe, unknown* |
| 15.3 | Visual? | *No, some, blind, unknown* |
| 15.4 | Strabismus? | *No, yes, unknown* |
| 15.5 | Hearing? | *No, some, deaf, unknown* |
| 15.6 | Speech? | *No, some, nonverbal, unknown* |

| **16** | Variables for timing of CP | |
| --- | --- | --- |
|  | **Variable** | **Description** |
| 16.1 | Timing? | *Unknown, during pregnancy and up to first 28 days of life (pre & perinatal), after first 28 days of life (postnatal)* |

| **17** | Variables for confirmed cause of CP | |
| --- | --- | --- |
|  | **Variable** | **Description** |
| 17.1 | Cause? | *Unknown, in utero cytomegalovirus, other infection (toxoplasmosis, rubella, herpes simplex virus), other infection *, other cause ** |
| 17.2 | Head injury? | *RTA, non-accidental, fall, other ** |
| 17.3 | Infection? | *Unspecified cause, viral, bacterial, dehydration due to gastroenteritis* |
| 17.4 | Stroke or CVA? | *During or following surgery, spontaneous, associated with other cardiac complications* |
| 17.5 | Other? | *Post seizure, near sudden infant death syndrome (SIDS), post immunisation, near drowning, peri-operative hypoxia, apparent life-threatening event, other ** |
| * Details to be given in the ‘Comments’ variable. | | |

| **18** | Variable – comments | |
| --- | --- | --- |
|  | **Variable** | **Description** |
| 18.1 | Comments | *This field for further comments or details.* |
