# Supplementary material for: Saudi Cerebral Palsy Register (SCPR): Protocol on the Methods and Technical Details
Source: J Epidemiol Glob Health. 2024 Feb 15;14(2):453–61. doi: 10.1007/s44197-024-00198-5 (PMC11176122; doi:10.1007/s44197-024-00198-5)
Supplement: Supplementary file 3 — Supplementary file3 (DOCX 33 KB) [file 44197_2024_198_MOESM3_ESM.docx]

|  |  |
| --- | --- |
| **1. ما هو السجل السعودي للشلل الدماغي؟** | **1. What is the Saudi Cerebral Palsy Register?** |
|  |  |
| السجل السعودي للشلل الدماغي هو قاعدة بيانات للمعلومات السريرية حول الشلل الدماغي. تتضمن المعلومات التي يتم جمعها عن كل شخص مصاب بالشلل الدماغي التاريخ العائلي، وتاريخ الحالات الطبية قبل الحمل، وتفاصيل حول الحمل، وصلة القرابة، والعيوب الخلقية، وتفاصيل الولادة، ونوع وشدة الشلل الدماغي، والإعاقات الأخرى المرتبطة به، والتركيبة السكانية للوالدين. يهدف السجل إلى تقديم صورة وطنية للشلل الدماغي. تم إطلاق السجل في عام 0000ه وسيكون أحد أهم السجلات النشطة للشلل الدماغي في العالم.  يُطلب من الأشخاص المصابين بالشلل الدماغي وأسرهم التسجيل عن طريق أقرب مركز إعادة تأهيل لهم. | The SCPR is a database of clinical information about cerebral palsy (CP). Information collected about each person with CP includes family history, history of medical conditions before pregnancy, details about the pregnancy, consanguinity, gestation, birth defects, birth details, type and severity of CP, other associated impairments and parent demographics. The register aims to provide a national picture of CP. The SCPR was launched in 0000 and will be one of the most important active CP registers in the world.  People with CP and their families are asked to register through the nearest rehabilitation centre. |
|  |  |
| **2. لماذا الحاجة إلى سجل للشلل الدماغي؟** | **2. Why is the SCPR needed?** |
|  |  |
| الشلل الدماغي هو اضطراب في الحركة والوضعية ناتج عن إصابة في الدماغ خلال المراحل المبكرة للنمو. إنه السبب الأكثر شيوعًا للإعاقة الجسدية في الطفولة. لا يوجد علاج معروف للشلل الدماغي كما ظلت المعدلات غير معروفة على المستوى الوطني مع عدم وجود إحصائيات دقيقة تخص الشلل الدماغي. من المعروف الآن أن معظم الشلل الدماغي يحدث نتيجة لعوامل معظمها قبل الولادة، إلا أنه لا يُعرف الكثير عن هذه العوامل.  يوفر السجل نظامًا منسقًا لجمع البيانات عن الشلل الدماغي في المملكة، وسيزيد من نطاق البحث في الشلل الدماغي. سيؤدي إلى تحسين جودة البحث من خلال توفير الوصول إلى عينة كبيرة من السكان. | CP is a disorder of movement and posture resulting from injury to the developing brain. It is the most common cause of physical disability in childhood. There is no known cure for CP and rates remain unknown nationally, with no accurate statistics available about CP. It is now recognised that most CP occurs as a result of factors present before birth, however little is known about such factors.  The SCPR provides a coordinated data collection system for CP in the Kingdom, and will increase the scope of research into CP. It will improve research quality by providing access to a large population sample. |
|  |  |
| **3. ما هي أهداف السجل؟** | **3. What are the aims of the register?** |
|  |  |
| الأهداف الرئيسية للسجل هي: | The main aims of the register are to: |
| - المساعدة في التخطيط للخدمات المقدمة للأشخاص المصابين بالشلل الدماغي | - Assist in planning services for people who have CP |
| - رصد ومراقبة الشلل الدماغي | - Surveillance and monitor trends of CP |
| - الحصول على المزيد من الفهم حول أسباب الإصابة بالشلل الدماغي | - Gain further understanding about the causes of CP |
| - تطوير وتقييم الاستراتيجيات الوقائية | - Develop and evaluate preventative strategies |
|  |  |
| سيوفر السجل إطارًا وطنيًا للبحث. ستساعد المعلومات الواردة في السجل في المشاريع البحثية التي تركز على أسباب الشلل الدماغي والوقاية منه. بالإضافة إلى ذلك ، سيوفر السجل تقارير للجهات الحكومية ومقدمي الخدمات. سيمكّن هذا من التخطيط بشكل أفضل للاحتياجات والخدمات الحالية والمستقبلية للأشخاص المصابين بالشلل الدماغي. | The SCPR will provide a national framework for research. The information contained on the register will assist with research projects that focus on causes, prevention and management of CP. In addition, the register will generate reports on demographics, frequency, distribution and severity of CP for government and service providers. This will enable better planning for the present and future service provision needs of people with CP. |
|  |  |
| **4. ما هي المعلومات التي سوف يتم جمعها؟** | **4. What information will be collected?** |
|  |  |
| لتقديم أفضل رعاية للأشخاص المصابين بالشلل الدماغي، نقوم بجمع أنواع مختلفة من المعلومات الصحية. يتضمن ذلك التفاصيل الأساسية مثل العمر والمكان الذي يعيشون فيه ، بالإضافة إلى التاريخ الطبي والعلاجات التي تلقوها. نقوم أيضا بجمع معلومات مفصلة عن التاريخ العائلي والطبي لفهم أي عوامل استقلابية أو وراثية مرتبطة بالشلل الدماغي. | To give the best care to people with cerebral palsy, we collect different types of health information. This includes basic details like age and where they live, as well as medical history, and treatments received. We also gather detailed family and medical history information to understand any metabolic or genetic factors related to cerebral palsy. |
|  |  |
| **5. كيف يمكنني المشاركة؟** | **5. How can I participate?** |
|  |  |
| يمكن للأشخاص المصابين بالشلل الدماغي وأسرهم التسجيل بعدة طرق: | People with CP and their families can register in a number of ways: |
| - قم بتسجيل الدخول إلى موقع السجل على الانترنت www.???.???.sa وأكمل المعلومات المطلوبة بنفسك | - Log in to the CP Register website www.???.???.sa and submit the required information yourself |
| - تواصل مع أقرب مركز إعادة تأهيل | - Contact your nearest rehabilitation centre |
| - امنح الإذن للممارس الصحي أو أخصائي التأهيل الخاص بك لتسجيل بياناتك | - Give permission for your health practitioner or education professional to register your details |
|  |  |
| سيتم الاتصال بالأطفال الذين تقل أعمارهم عن 5 سنوات مرة أخرى بعد بلوغهم السنة الخامسة من العمر وذلك للتأكيد النهائي للتفاصيل. يرجى التواصل مع السجل إذا تغيرت بيانات التواصل الخاصة بكم. | Children under the age of 5 years will be contacted again after they reach the age of 5 for final confirmation of details. Please notify the register if your contact details change. |
|  |  |
| **6. ماذا عن الموافقة وسحب الموافقة؟** | **6. What about consent and withdrawal of consent?** |
|  |  |
| التسجيل طوعي. إذا كنت ترغب في تضمين التفاصيل الخاصة بك في السجل، فسيُطلب منك تقديم موافقة خطية. لا يمكن إدراج بياناتك في السجل حتى يتم استلام نموذج موافقة موقعة (ورقياً) من قبل مركز إعادة التأهيل الخاص بك. يمكنك تنزيل نموذج الموافقة من رابط التنزيل أو التواصل مع مركز إعادة التأهيل الخاص بك وسيقومون بتزويدك بالنموذج. | Registration is voluntary. If you would like to have your details included on the SCPR you will be asked to provide written consent. Your details cannot be included on the register until a signed consent form (paper) is received by your rehabilitation centre. You can download the consent form from download link or contact your rehabilitation centre and they will send one to you. |
|  |  |
| هناك مستويات مختلفة من الموافقة في السجل. سيُطلب منك الموافقة على: | There are different levels of consent on the register. You will be asked to consent to: |
| **أ)** جمع وتسجيل وتخزين المعلومات في السجل عن طريق مركز إعادة التأهيل الخاص بك. قد يشمل ذلك معلومات الولادة والسجلات الطبية الحالية * | **a)** The collection, recording and storage of information on the register by your rehabilitation centre. This may involve consulting birth and current medical records* |
| **ب)** نقل المعلومات مجهولة الهوية إلى السجل السعودي للشلل الدماغي | **b)** The transfer of de-identified information to the Saudi Cerebral Palsy Register |
| **ج)** تلقي دعوات من موظفي السجل للمشاركة في المشاريع البحثية | **c)** Receiving invitations from register staff to participate in research projects |
| **د)** يتم الاتصال بالممارسين الصحيين الذين ترشحهم للمساعدة في استكمال معلومات التسجيل والتحقق منها * | **d)** Health professionals that you nominate being contacted to assist in completing and verifying the register information * |
|  |  |
| يمكنك اختيار مساهمتك في السجل عن طريق تحديد مستوى موافقتك. يمكنك تغيير مستوى موافقتك في أي وقت عن طريق إرسال نموذج موافقة معدّل إلى السجل عن طريق أقرب مركز تأهيل. إذا غيرت رأيك في المستقبل بشأن المشاركة في السجل، فيمكنك سحب الموافقة وسيتم حذف اسمك من السجل. هذا لن يضر بك بأي شكل من الأشكال ولن يؤثر على الخدمات التي تتلقاها. | You can choose your contribution to the register by selecting your level of consent. You can change your level of consent at any time by submitting a revised consent form via nearest rehabilitation centre. If, in the future, you change your mind about participating in the register, you can withdraw consent and your name will be removed from the register. This will not disadvantage you in any way or affect services you are receiving. |
|  |  |
| *** يرجى التسجيل حتى إذا كنت لا تعرف إجابات جميع الأسئلة الموجودة في النموذج. سنبذل قصارى جهدنا في المساعدة لإكمال هذه المعلومات.** | *** Please register even if you do not know the answers to all of the questions on the form. We will do our best to help complete this information.** |
|  |  |
| **7. ماذا عن الخصوصية والسرية؟** | **What about privacy and confidentiality?** |
|  |  |
| نحن نحترم خصوصيتك. المعلومات التي تقدمها إلىى السجل سرية للغاية ولن تتم مشاركتها مع أي شخص. فقط موظفو السجل في مركز إعادة التاهيل الخاص بك هم من يمكنهم التعامل مع هذه البيانات. سيتم فقط تضمين بياناتك مجهولة الهوية في السجل. أي أبحاث أو تقارير يتم إنشاؤها من السجل أو المعلومات المقدمة للباحثين الآخرين وأفراد الجمهور ستكون خالية من جميع معلومات التعريف الشخصية. | We respect your privacy and take your confidentiality seriously. The data you provide will not be shared with anyone. The information you provide to the register is strictly confidential and will only be seen by register staff from your rehabilitation centre. Only your de-identified data will be included in the SCPR. Any research or reports generated from the register or information provided to other researchers and members of the public will be free of personal identifying information. |
|  |  |
| إذا وافقت ، فقد يتصل بك موظفو السجل من وقت لآخر بخيار المشاركة في دراسة بحثية. سيقوم موظفو السجل بإرسال معلومات الدراسة الخاصة بك ويمكنك بعد ذلك الاتصال بالباحثين مباشرة إذا كنت ترغب في المشاركة أو إذا كنت بحاجة إلى مزيد من المعلومات. الموافقة على المشاركة في هذه الأبحاث أو رفضها هو قرارك الشخصي. لن يقدم السجل بياناتك الشخصية أو بيانات الاتصال للباحثين أو أي شخص آخر مطلقاً. | If you consent, from time to time CP Register staff may contact you with the option of participating in a research study. Register staff will forward you study information and you can then contact the researchers directly if you would like to participate or if you require more information. It is your decision to agree to or decline these offers. The CP Register will never provide your personal or contact details to researchers or anyone else. |
|  |  |
| **ورقة المعلومات هذه لك للاحتفاظ بها. إذا كانت لديك أي أسئلة أو ترغب في معرفة المزيد عن هذا المشروع، يرجى التواصل مع مركز إعادة التأهيل الخاص بك.** | **This information sheet is for you to keep. If you have any questions or would like to know more about this project, please contact your rehabilitation centre.** |
|  |  |
|  |  |
| **للمزيد من المعلومات يرجى التواصل معنا عن طريق إحدى القنوات التالية:** | **For more information, please contact us through one of the following channels:** |
|  |  |
| **هاتف**: 000 000 0000 | **Phone**: 000 000 0000 |
| **بريد الكتروني**: ???@??? | **Email**: ???@??? |
|  |  |
| **أو تفضل بزيارة موقعنا على الشبكة العنكبوتية:**  www.???.???.sa | **Or visit our website:**  www.???.???.sa |

| السجل السعودي للشلل الدماغي | شعار السجل  SCPR LOGO | Saudi Cerebral Palsy Register |
| --- | --- | --- |
| هيئة الصحة العامة |  | Public Health Authority |
| 7090 طريق الملك عبدالعزيز – حي العارض |  | 7090 King Abdulaziz Rd – Al Arid Dist. |
| الرياض 13351 – 5210 |  | RIYADH 13351 – 5210 |
| المملكة العربية السعودية |  | Kingdom of Saudi Arabia |
